# Protective effects of lipoic acid on chrysene-induced toxicity on Müller cells in vitro

**Published:** 2013-01-07

**Authors:** Saffar Mansoor, Navin Gupta, Georgia Luczy-Bachman, G. Astrid Limb, Baruch D. Kuppermann, M. Cristina Kenney

**Affiliations:** 1 From the Gavin Herbert Eye Institute, School of Medicine, University of California, Irvine, CA; 2 Department of Pediatrics, Clinical Translational Science Center, University of California, Irvine, CA; 3 Department of Ocular Biology and Therapeutics, UCL, Institute of Ophthalmology, London, United Kingdom

## Abstract

**Purpose:**

This study evaluates the toxic effects of chrysene (a component from cigarette smoke) on Müller cells (MIO-M1) in vitro and investigates whether the inhibitor lipoic acid can reverse the chrysene-induced toxic effects.

**Methods:**

MIO-M1 cells were exposed to varying concentrations of chrysene with or without lipoic acid. Cell viability was measured by a trypan blue dye exclusion assay. Caspase-3/7 activity was measured by a fluorochrome assay. Lactate dehydrogenase (LDH) release was quantified by an LDH assay. The production of reactive oxygen/nitrogen species (ROS/RNS) was measured with a 2’,7’-dichlorodihydrofluorescein diacetate dye assay. Mitochondrial membrane potential (ΔΨm) was measured using the JC-1 assay. Intracellular ATP content was determined by the ATPLite kit.

**Results:**

MIO-M1 cells showed significantly decreased cell viability, increased caspase-3/7 activity, LDH release at the highest chrysene concentration, elevated ROS/RNS levels, decreased ΔΨm value, and decreased intracellular ATP content after exposure to 300, 500, and 1,000 µM chrysene compared with the control. Pretreatment with 80 µM lipoic acid reversed loss of cell viability in 500-µM-chrysene-treated cultures (24.7%, p<0.001). Similarly, pretreatment with 80 µM lipoic acid before chrysene resulted in decreased caspase-3/7 activities (75.7%, p<0.001), decreased ROS/RNS levels (80.02%, p<0.001), increased ΔΨm values (86%, p<0.001), and increased ATP levels (40.5%, p<0.001) compared to 500-µM-chrysene-treated cultures.

**Conclusions:**

Chrysene, a component of cigarette smoke, can diminish cell viability in MIO-M1 cells in vitro by apoptosis at the lower concentrations of Chrysene (300 and 500 µM) and necrosis at the highest concentration. Moreover, mitochondrial function was particularly altered. However, lipoic acid can partially reverse the cytotoxic effect of chrysene. Lipoic acid administration may reduce or prevent Müller cell degeneration in retinal degenerative disorders.

## Introduction

Age-related macular degeneration (AMD) is the leading cause of vision loss in the aging population in the Western world [1]. The prevalence of the disease is expected to increase in the coming years as people live longer, and this calls for a better understanding of the mechanisms involved in AMD. The early clinical presentation of the disease is altered pigmentation and/or yellowish subretinal deposits known as drusen in the macula. Over time, drusen may become confluent and lead to degeneration of retinal pigment epithelium (RPE) cells and/or photoreceptors (dry form). In the wet form of AMD, growth of choroidal blood vessels into the retina occurs, which is referred to as choroidal neovascularization (CNV). Both forms of AMD can have damaging effects on central visual function [2].

The pathogenesis of AMD is still unknown, but multiple studies have linked cigarette smoking to an increased risk of AMD development [3-5]. A twofold to fourfold increase risk of AMD has been found in smokers as compared with nonsmokers [4-6]. Cigarette smoking has been associated with the development of both the wet form of AMD as suggested in the macular photocoagulation study of 1986 [7] as well as the late, dry form or geographic atrophy [2,6,8].

Although cigarette smoke contains over 4,000 chemicals, polycyclic aromatic hydrocarbons (PAH) are the most toxic substances known to be present in cigarette smoke. Chrysene is one of the PAHs found in cigarette smoke. Each cigarette delivers approximately 60 ng of chrysene (Speclab) [9]. However, it is difficult to ascertain the quantitative level of chrysene because of variability in smoking devices, such as cigarettes (which come in various sizes), cigars, pipes stuffed with tobacco or hookas/beedies (raw tobacco) used in old world cultures, frequency of smoking, average inhalation, the concentration ultimately inhaled, amount of chrysene (from smoke) reaching systemic circulation, and the quantity cross through the blood–retinal barrier to reach into the retina. In addition, chrysene is a soil and water contaminant and also occurs as a ubiquitous environmental pollutant from smoked foods, coal gasification, road and roof tarring, incinerators, and aluminum production (IARC) [10,11].

In vitro and in vivo studies have shown that PAH can have chemical effects via formation of DNA adducts, which lead to cellular proliferation [12-14]. Chrysene or its derivative have mutagenic, carcinogenic [15], and genotoxic [16,17] effects in animal and cell culture studies. Chrysene caused alteration in immune function and CYP450 activity in adult male deer mice (*Peromyscus maniculatus*) [18]. In addition, chrysene showed toxic effects in rat liver epithelial cells [19] and in marine organisms [20].

In smokers, retinal tissues that can be damaged secondary to degeneration of RPE cells and Bruch’s membrane are the neurosensory cells (photoreceptors) and supportive glial cells [21,22]. Postmortem eyes of patients with AMD have shown death of photoreceptors, inner nuclear layer, and RPE cells [23]. As of yet, there are few studies on the effects of chrysene in human retinal cells. Our overall goal is to investigate the pathophysiology of cigarette smoke in AMD. Investigators have examined other smoking toxicants, such as benzo-e-pyrene (BeP) [24,25] and nicotine [26] with and without various inhibitors that can reverse their toxic effects. Nicotine treatment showed a differential response of cells ranging from not being affected at all (ARPE-19 cells) to loss of cell viability via necrosis (human microvascular endothelial cells [HMVEC]) or cell damage through an oxidant pathway (R28 cells) [26]. Jia et al. reported that acrolein, a toxicant in cigarette smoke, causes oxidative damage and mitochondrial dysfunction in RPE cells which was exposed to the protective agent alpha-lipoic acid (LA) [27]. These findings underscore the challenges in developing effective inhibitor therapies to reverse smoking-related cell damage. We are now expanding our studies to include human retinal glial cells and their response to smoking-related compounds.

Inhibitors studied in our laboratory are memantine, epicatechin, resveratrol, and genistein, all of which showed significant inhibitory effects against smoking constituents and other toxins, such as ketocholesterol [25,26,28]. Recently, LA has shown great promise in protecting cells against various toxicants, including those from smoke [29,30]]. LA is a sulfur-containing compound found naturally in plants and animals. Dihydrolipoic acid is the reduced form, which is the form that exists intracellularly. It is present in mitochondria as an essential cofactor for pyruvate dehydrogenase and α-ketoglutarate dehydrogenase [31]. LA can scavenge hydroxyl radicals, singlet oxygen, peroxynitrite, and nitric oxide [31,32]]. In addition it can chelate several transition metal ions [31,33]], has antioxidant properties, and is reported to provide protection against oxidative injury in various disease process, including neurodegenerative disorders and diabetic syndrome [34,35]]. This study investigates whether chrysene causes injury, especially mitochondrial dysfunction, to Müller cells and whether LA would protect it from chrysene-mediated injury and mitochondrial dysfunction.

## Methods

### Cell culture and treatments

The human Müller cell line (MIO-M1) [36] was grown in Dulbecco’s modified Eagle medium (D-MEM; 1×) high glucose SKU#10569–044 (GlutaMAX-1 medium substituted on a molar equivalent basis for L-glutamine, 4500 mg/l D-glucose, 110 mg/l sodium pyruvate, 1× penicillin/streptomycin, and 10% fetal bovine serum). MIO-M1 cells that were used for these series of experiments were of passage 26 to 30. Cells were plated in 6-, 96-, or 24-well plates (Becton Dickinson Labware, Franklin Lakes, NJ) for cell viability (5×10^5^ cells/well), caspase-3/7 activity (1.2×10^5^ cells/well), lactate dehydrogenase (LDH; 1.2×10^5^ cells/well), mitochondrial membrane potential (1.2×10^5^ cells/well), reactive oxygen/nitrogen species (ROS/RNS) detection (1.2×10^5^ cells/well), and ATP measurement (1.0×10^5^ cells/well) assays and were incubated at 37 °C in 5% CO_2_ until monolayer confluence was achieved. Cells were incubated further in serum-free culture medium for 24 h to make them relatively nonproliferating. This was done to simulate the condition of natural human retina in which Müller cells remain in a nonproliferating phase and are not exposed to the circulation because of the blood–retinal barrier [24].

### Exposure to chrysene

Chrysene was procured from Sigma Aldrich Inc. (St Louis, MO), and the stock solution (100 mM chrysene) was prepared by solubilizing 0.0228 gm of chrysene in 1 ml of dimethyl sulfoxide (DMSO). Thereafter, different dilutions were prepared by adding to the culture media. Cells were treated with 1,000 µM, 500 µM, 300 µM, and 100 µM chrysene for 24 h. Some other concentrations of chrysene (5, 10, 20, 30, and 40 µM) were also used to treat cells for 24 h or 7 days. Cells with equivalent DMSO (without chrysene) served as control cultures.

### Cell viability studies

MIO-M1 cells were treated with chrysene in three different ways: (a) acute exposure with 10, 20, 30, and 40 µM chrysene for 24 h, (b) chronic exposure with 5, 10, 20, and 30 µM chrysene for 7 days, and (c) treatment with 100, 300, 500, and 1,000 µM chrysene for 24 h. The cell viability (CV) assay was performed as described by Narayanan et al. [37]. Cells were harvested from the six-well plates by treatment with 0.2% trypsin–EDTA and then incubated at 37 °C for 5 min. The cells were centrifuged at 3920 × *g* for 5 min and resuspended in 1 ml of culture medium. CV was analyzed by a Vi-cell series cell viability analyzer (Beckman Coulter Inc., Fullerton, CA). The analyzer performs an automated trypan blue dye-exclusion assay and gives the percentage of viable cells.

### Inhibition studies with lipoic acid

To examine inhibitory effects on loss of CV, cells were pretreated for 6 h with different concentrations of R-alpha-LA (Sigma Aldrich Inc.) and then replaced with chrysene+LA. LA was dissolved in distilled water and prepared as 10, 20, 40, 80, or 100 µM in culture media. Chrysene was added to the pretreated cells, which were then cultured overnight and analyzed for CV. The higher percentage of viable cells in the pretreated cultures indicated a greater inhibitory effect. Therefore, the optimum inhibitory effect of LA at a particular concentration was determined on the basis of percentage viable cells.

### Caspase-3/7 assays

To verify apoptosis as a cell death mechanism, caspase-3/7 activity was detected using fluorescent-labeled inhibitor of caspases apoptosis (FLICA) detection kits (Immunochemistry Technologies LLC, Bloomington, MN). The FLICA reagent has an optimal excitation range from 488 to 492 nm and an emission range from 515 to 535 nm. Caspase-3/7 activities were measured using a fluorescence image scanning unit instrument (FMBIO III; Hitachi, Yokohama, Japan), which quantified apoptosis as the amount of green fluorescence emitted from FLICA probes bound to caspase-3/7.

At the designated time period, the wells were rinsed briefly with fresh culture media, replaced with 300 µl/well of 1× FLICA solution in culture media, and incubated at 37 °C for 1 h under 5% CO_2_. Cells were washed with PBS (8g NaCl, 0.2g KCl, 1.15 g Na_2_HPO_4_, 0.2g KH_2_PO_4_ dissolved in one liter dionized water). Non-apoptotic cells appeared unstained, while cells undergoing apoptosis fluoresced brightly. The following controls were included: untreated MIO-M1 cells without FLICA to exclude auto fluorescence from MIO-M1 cells; untreated MIO-M1 cells with FLICA for comparison of caspase activity of treated cells; tissue culture plate wells without cells with buffer alone to represent the background level; tissue culture plate wells without cells with buffer and DMSO to exclude the cross-reaction of FLICA with DMSO and culture media; MIO-M1 cells with DMSO and FLICA to account for any cross-fluorescence between untreated cells and DMSO. Quantitative calculations of caspase activities were performed with an FMBIO III (Hitachi). The caspase activity was measured as the average signal intensity of the fluorescence of the pixels in a designated spot (mean signal intensity [msi]).

### DNA fragmentation assay

MIO-M1 cells (5×10^5^) were plated overnight in six-well plates and then incubated for another 24 h with different concentrations of chrysene. DNA was extracted (QIAamp DNA Micro kit; Qiagen, Hilden, Germany) according to the manufacturer’s instructions. Briefly, the cells in the experiment were first lysed. The lysate was then transferred onto the kit columns. As the columns were centrifuged, the DNA present in the lysate was adsorbed onto-the silica gel membrane. While DNA remained bound to this silica membrane, the columns were made to get rid of contaminants by washing away with buffers AW1 and AW2. DNA was then eluted from the columns using distilled water. Samples were separated by electrophoresis on 3% agarose gels and were stained with 5% ethidium bromide. 100 bp DNA step ladder marker from Promega was used, and images were captured with an FMBIO III (Hitachi).

### Lactate dehydrogenase cytotoxicity assay

LDH is a cytosolic enzyme present in all mammalian cells and is released following damage of the plasma membrane. Therefore, LDH is the marker of cell death, and this assay is a test for cytotoxicity in vitro. The activity of released LDH in culture supernatant was measured with a commercial LDH-Cytotoxicity Assay Kit II (BioVision Research Products, Mountain View, CA). The basis of this kit is a coupled enzymatic reaction in which LDH present within the sample catalyzes the conversion of lactate into pyruvate with the concomitant formation of nicotinamide adenine dinucleotide (NADH) from NAD^+^. The NADH is then used as a cofactor in the conversion of the tetrazolium salt, 2-p-iodophenyl-3-p-nitrophenyl tetrazolium chloride, into a red formazan product; this second reaction is catalyzed by the enzyme diaphorase, which is present within the assay substrate mixture. The absorbance of the formazan product is measured at 490 nm. Formazan concentrations are directly proportional to the concentration of LDH in the sample.

The LDH cytotoxicity assay was performed following the supplier’s protocol. Briefly, MIO-M1 cells were plated with 100 µl culture medium in each well in 96-well plates (in triplicate).There were untreated controls and background controls having the same volume of culture medium per well with and without cells, respectively. After chrysene treatment, the 96-well plate was shaken gently for even distribution of LDH in the culture medium. The cells were centrifuged at 58,800 × *g* for 10 min to precipitate the cells. Then 50 µl of supernatant was transferred into a new 96-well plate, which was treated with 100 µl/well of LDH reaction mixture (LDH reaction mixture was prepared by mixing the water-soluble terazolium (WST) substrate mix with LDH assay buffer). After 30-min incubation at room temperature, the LDH activity was quantified as absorbance values (optical density [OD]) at 490 nm by a multiwell spectrophotometer (Victor 2 microplate reader; Perkin Elmer, Wellesley, MA). The plate was read at multiple time points until consistent readings were obtained. LDH activity in each cell lysate was expressed as percent increase in LDH activity with respect to equivalent DMSO control.

### Detection of reactive oxygen/nitrogen species production

ROS/RNS production was measured with the fluorescent dye 2’-,7’-dichlorodihydrofluorescein diacetate assay (H_2_DCFDA; Invitrogen, Molecular Probes, CA) [38], which detects hydrogen peroxide, peroxyl radicals, and peroxynitrite anions. The cells were washed with sterile PBS and incubated with 500 µl of 10 µM H_2_DCFDA for 30 min at 37 °C and again washed with PBS. The H_2_DCFDA (10 µM) was prepared by adding 2 µl of 5 mM (H_2_DCFDA) stock/ml in serum-free culture media. The 5 mM H_2_DCFDA stock solution was prepared fresh by mixing 0.005 g of H_2_DCFDA in 2.05 ml of DMSO. ROS/RNS production was measured with the scanning unit (excitation 488 nm, emission 520 nm; FMBIO III; Hitachi).

### Mitochondrial membrane potential assay

Loss of the ΔΨm is a hallmark for cellular apoptosis and was measured using the JC-1 mitochondrial membrane potential detection kit (Biotium, Hayward, CA). JC-1 contains a cationic dye (5,5′,6,6’-tetrachloro-1,1’,3,3′-tetraethyl-benzimidazolyl-carbocyanine-iodide) that fluoresces red in the mitochondria of live cells. In dead cells the mitochondrial membrane potential collapses and the cationic dye remains in the cytoplasm and fluoresces green. Typically, the ratio of red to green fluorescence is higher in healthy cells and comparatively lower in apoptotic cells.

The JC-1 assay was conducted as per the supplier’s instructions. Briefly, at the end of the 24 h of chrysene with or without lipoic acid exposure, the cells were rinsed with fresh media and incubated for 15 min with 500 µl/well of JC-1 reagent in culture media. Images were captured using an FMBIO III instrument (Hitachi) and the red/green fluorescence ratios were calculated.

### Measurement of intracellular ATP

Intracellular ATP level was measured using the luminescence ATP detection assay (ATPlite PerkinElmer Inc., Waltham, MA) as per the supplier’s instruction. ATPlite assay is based on production of light caused by the reaction of ATP with added luciferase and D-luciferin (just like the firefly luciferase). The emitted light is proportional to the ATP concentration. Cells were first plated in the 96-well culture plate. Cell lysis solution was added to the wells to lyse the cells and release the ATP. Exposure time was 5 min Luciferase and D-luciferin is then added to it. Exposure time was 5 min. Then the plate was dark adapted for 10 min. The luminescence is then measured with the reader.

ATP is a marker for cell viability. Its concentration in the cell declines rapidly when the cell dies either due to necrosis or apotosis. This assay is based on light production caused by the reaction of ATP with added luciferase and D-luciferin. The emitted light is proportional to the ATP concentration within certain limits.

Briefly, MIO-M1 cells were first plated on 96-well plates (100,000 cells per 100 µl culture media/well) and were incubated overnight. They were pretreated with different concentrations of chrysene. To each well, 50 µl of mammalian cell lysis solution was added, which opens up the cells allowing the intracellular ATP to be released. To each well, 50 µl of ATPlite buffer (HEPES (4-(2-hydroxyethyl)-1-piperazineethanesulfonic acid) is a zwiterronic organic chemical buffering agent) was added, which contained luciferase and D-lucifern. The microplate was then shaken for 5 min at 6860 × *g*. The plate was covered with an adhesive seal, dark-adapted for 10 min, and luminescence was measurement using a luminescence microplate reader (BioTek Instrument, Inc., Winooski, VT). The ATP standard curve was made by plotting signal verses ATP concentrations. The signal for the unknown sample was obtained by using a linear regression equation.

### Statistical analysis

Data were subjected to statistical analysis by ANOVA (Prism, version 3.0; GraphPad Software Inc., San Diego, CA). Newman–Keuls multiple-comparison test was done to compare the data within each experiment. A value of p<0.05 was considered statistically significant. Error bars in the graphs represent the standard error of the mean with experiments performed in triplicate.

## Results

### Cell viability studies

**Figure 1:**
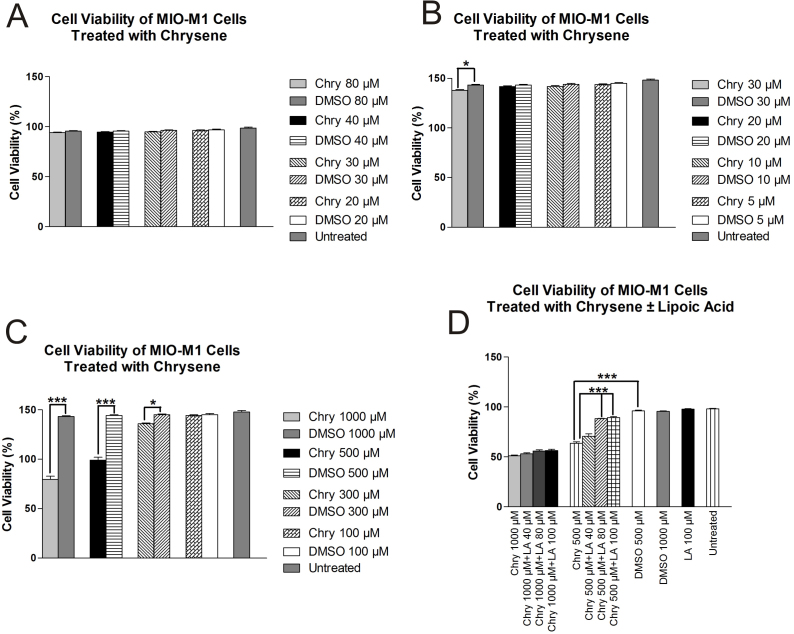
The effects of chrysene on MIO-M1 cell viability. **A**: There were no changes of MIO-M1 cell viability after 24 h exposure to 20, 30, 40, and 80 μM chrysene (Chry) compared to the dimethyl sulfoxide (DMSO)-equivalent controls. **B**: After exposure to 30 μM chrysene for 7 days, the MIO-M1 cells showed a decrease of cell viability compared to DMSO-equivalent cells (*p<0.05). The cell viabilities in MIO-M1 cells exposed to 5, 10, and 20 μM chrysene for 7 days were similar to DMSO-equivalent controls and untreated controls. **C**: There were significant decreases of MIO-M1 cell viabilities after 24 h treatment with 1,000 μM (***p<0.001), 500 μM (***p<0.001), and 300 μM (*p<0.05) chrysene compared to DMSO-equivalent controls. Cultures treated with 100 μM chrysene showed a similar level of cell viability to DMSO-equivalent controls. **D**: Some cells were pretreatment 6 h with varying concentrations of lipoic acid (LA) and then exposed to 500 μM or 1,000 μM chrysene (Chry +LA) for an additional 24 h. Cell viability levels were not reversed by LA pretreatment in any of the MIO-M1 cells exposed to 1,000 μM chrysene. The loss of cell viability was reversed by pretreatment with 80 μM LA (p<0.001) and 100 μM LA (p<0.001) in the MIO-M1 cells exposed to 500 μM chrysene. Cell viability levels were not decreased in MIO-M1 cultures treated with 1,000 μM DMSO-equivalent, 500 μM DMSO-equivalent, or by exposure to 100 μM LA alone. Assays were performed in triplicate and the experiments repeated three times. Values are mean ± standard error mean (SEM).

MIO-M1 cells exposed to chrysene 20, 30, 40, and 80 µM for 24 h did not show toxic effects on cells (Figure 1A). However, cells treated with 30 µM chrysene for 7 days (chronic exposure) showed significantly reduced cell viability (91.85±0.6%, p<0.05) as compared to controls (95.45±0.55%); treatment with 5, 10, and 20 µM chrysene for 1 week did not affect cell viability on MIO-M1 cells (Figure 1B). MIO-M1 cells showed loss of CV after exposure to higher concentrations of chrysene for 24 h (Figure 1C). The mean percentage of viable cells was 53.1±2.1 (p<0.001), 66.1±1.9 (p<0.001), and 90.65±0.55 (p<0.05) at 1,000, 500, and 300 µM chrysene, respectively, compared to DMSO-treated controls. At 100 µM chrysene, CV was 96.3±0.5 (p>0.05). The mean percentages of cell viability of DMSO-treated equivalent cultures of 1,000 (95.56±0.5), 500 (96.15±0.65), 300 (96.45±0.6), and 100 (96.75±0.8) µM were similar to those of the untreated MIO-M1 cultures (98.5±1). Cell death induced by 1,000 µM chrysene was not protected by pretreatment with LA (40, 80, and 100 µM) at any of the studied concentrations (Figure 1D). The optimum increase in mean percentage of CV due to pretreatment with LA 80 µM was 4.85% (55.75±1.25, p>0.05) as compared to 1,000 µM chrysene alone (50.9±0.89). However, in the MIO-M1 cells exposed to 500 µM chrysene, pretreatment with 80 µM LA significantly increased the mean percentage of CV. The increase in mean percentage of CV was 24.7 (88.2±1.5, p<0.001) as compared to 500-µM-chrysene-treated cultures (63.5±2). Therefore, in further experiments cells were pretreated with 80 µM LA to examine the pathways for protective effects.

### Caspase-3/7 activity

**Figure 2:**
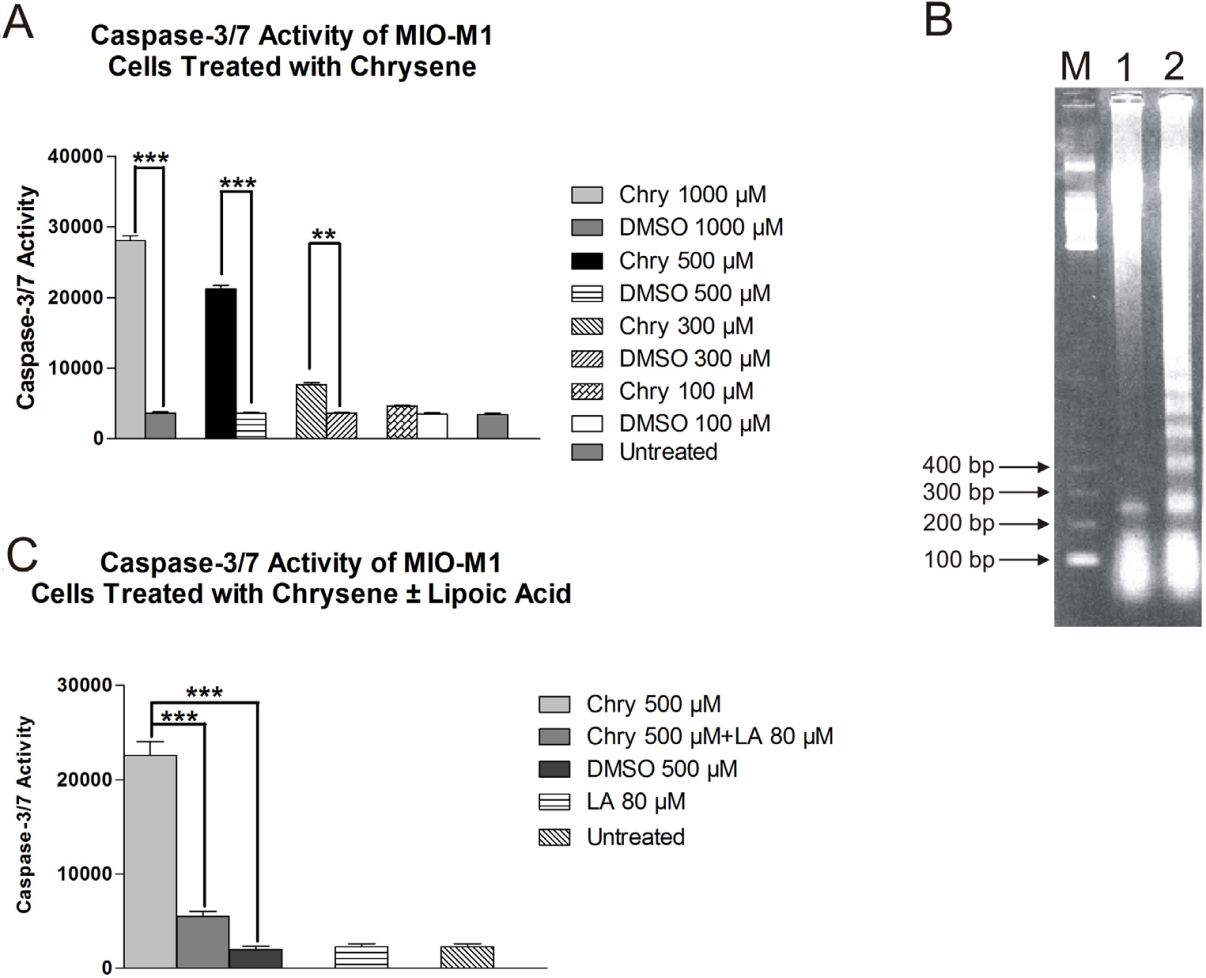
The effects of chrysene (Chry) treatment on caspase-3/7 activity in MIO-M1 cells. **A**: The MIO-M1 cells treated 24 h with 1,000 μM (***p<0.001), 500 μM (***p<0.001), and 300 μM (**p<0.01) chrysene showed significantly increased caspase-3/7 activities compared to dimethyl sulfoxide (DMSO)-equivalent-treated cells. The untreated cells, 100-μM-chrysene-treated cells, and equivalent-DMSO-treated cells showed similar levels of caspase-3/7 activity. **B**: Analyses of DNA fragmentation patterns for chrysene-treated MIO-M1 cells. After treatment with 100 μM chrysene (lane 2), the MIO-M1 cells showed DNA fragmentation at 200-base pair (bp) intervals compared to the untreated control cultures (lane 1) which is consistent with apoptosis. **C**: The protective effects of lipoic acid (LA) against chrysene induced caspase-3/7 activity in MIO-M1 cells. The MIO-M1 cells pretreated 6 h with 80 μM LA followed by the addition of 500 μM chrysene (500 μM +LA 80 μM) for 24 h showed lower caspase-3/7 activity levels compared to the cells treated with 500 μM chrysene alone (***p<0.001). The untreated cells, DMSO-equivalent-treated cells (DMSO 500 μM), and cells treated with 80 μM LA alone (LA 80 μM) showed low levels of caspase-3/7 activity. Assays were performed in triplicate and the experiments repeated three times. The DNA fragmentation analyses were repeated twice. Values are mean±standard error mean (SEM). M, marker; bp, base pair.

Caspase-3/7 activity in MIO-M1 cells increased significantly after treatment with chrysene for 24 h (Figure 2A). Cells treated with 1,000, 500, and 300 µM chrysene showed increase in mean fluorescence of 87% (28,066.70±698.4 msi, p<0.001), 83.7% (21,200±472.5 msi, p<0.001), and 52.5% (7666.67±296.27 msi, p<0.01), respectively. Cells treated with 100 µM chrysene did not show a significant increase in caspase-3/7 activity (4666.667±120.7 msi, p>0.05) as compared to DMSO-treated cultures (3500.0±173.2 msi). Values for untreated and DMSO-equivalent cultures of 1,000, 500, and 300 µM were 3416.667±187.8 msi, 3663.33±144.9 msi, 3630.33±124.9 msi, and 3639±123.2 msi, respectively. Caspase-3/7 is the hallmark of apoptosis because it is the final common pathway of apoptosis. To verify apoptotic activity, DNA fragmentation analysis was performed showing DNA bands that laddered in approximately 200-bp increments, consistent with apoptosis (Figure 2B). Pretreatment with 80 µM LA resulted in 75.7% (5466.6±578.3 msi, p<0.001) reduction in caspase-3/7 activity as compared to the 500-µM-chrysene-treated culture (22,566.67±1476.8 msi; Figure 2C).

### Lactate dehydrogenase cytotoxicity assay

**Figure 3:**
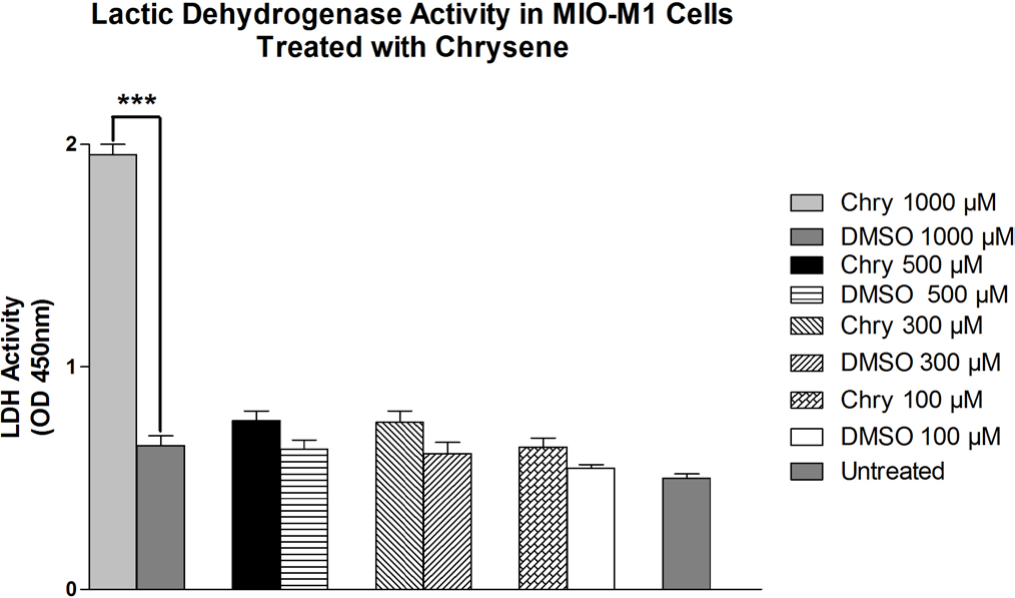
The effects of chrysene (Chry) on lactate dehydrogenase (LDH) levels in MIO-M1 cells. The MIO-M1 cells treated 24 h with 1,000 μM chrysene showed increased LDH levels activity (***p<0.001) compared to cells treated with the dimethyl sulfoxide (DMSO)-equivalent. The untreated cells, 500 μM-chrysene-treated cells, 300-μM-chrysene-treated cells, 100-μM-chrysene-treated cells, and their DMSO- equivalent-treated cells showed low levels of LDH. Assays were performed in triplicate and repeated three times. Assays were performed in triplicate and the experiments repeated three times. Values are mean ± standard error mean (SEM).

Treatment of MIO-M1 cells with 1,000 µM chrysene for 24 h resulted in a 67% OD (1.95±0.04, p<0.001) increase in LDH activity as compared to the DMSO-equivalent culture (OD 0.64±0.045; Figure 3). Chrysene at 500-µM (OD 0.76±0.04), 300-µM (OD 0.75±0.05), and 100-µM (OD 0.64±0.03)-treated MIO-M1 cells did not significantly increase LDH levels (p>0.05) compared to their equivalent DMSO controls (ODs 0.63±0.04, 0.61±0.05, and 0.054±0.01, respectively). The OD value for untreated MIO-M1 cells was 0.5±0.02.

### Reactive oxygen/nitrogen species measurement

**Figure 4:**
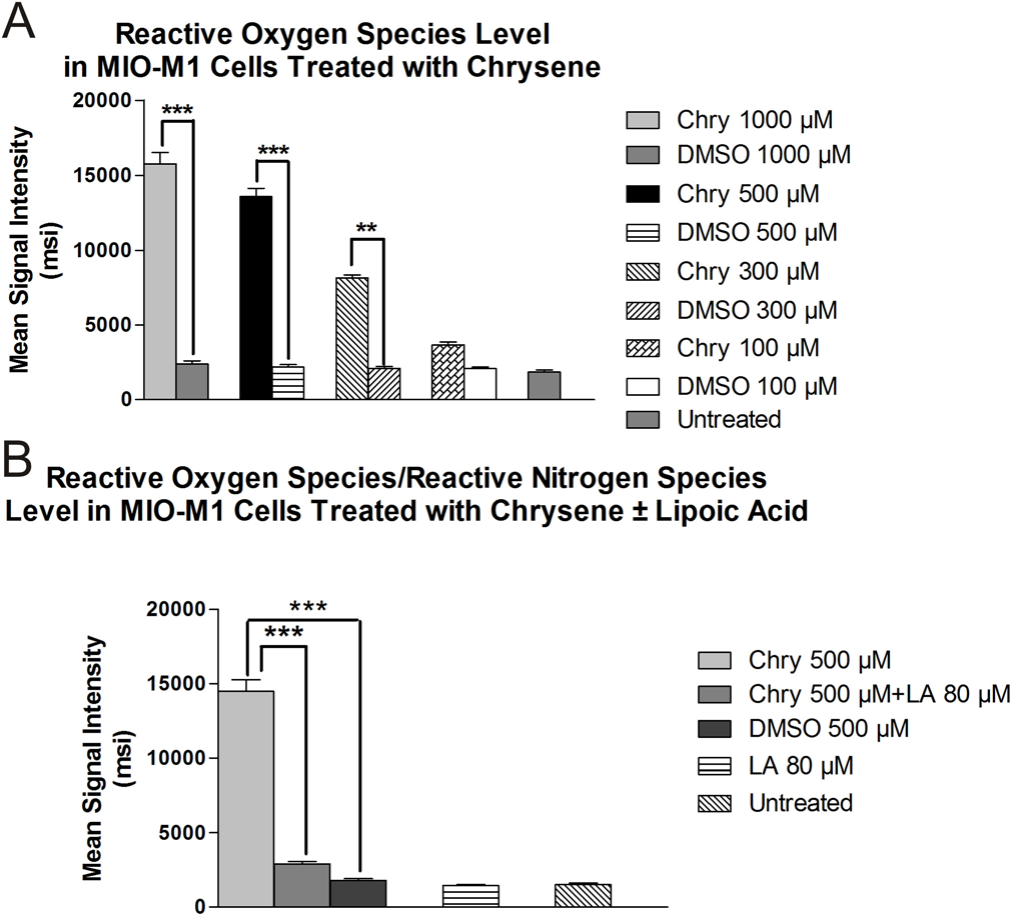
The effects of chrysene (Chry) on production of reactive oxygen/nitrogen species (ROS/RNS) in MIO-M1 cells. **A**: The MIO-M1 cells exposed to 1,000 μM chrysene, 500 μM chrysene, and 300 μM chrysene had significantly higher ROS/RNS compared to MIO-M1 cells treated with the dimethyl sulfoxide (DMSO)-equivalent (***p<0.001, ***p<0.001, **p<0.01, respectively). The untreated MIO-M1 cells, 100-μM-chrysene-treated cells, and DMSO-equivalent-treated cells showed low levels of ROS/RNS production. **B**: Cells that were pretreated 24 h with 80 μM lipoic acid (LA) and had 500 μM chrysene added to the cultures for 24 h (Chyr 500 μM +LA 80 μM) showed ROS/RNS levels similar to the DMSO- equivalent-treated cultures showing a protective effect of the LA against chrysene induced ROS/RNS production. Assays were performed in triplicate and the experiments repeated three times. Values are mean±standard error mean (SEM).

MIO-M1 cells treated for 24 h with 1,000, 500, and 300 µM chrysene showed significantly increased ROS/RNS levels as compared to the equivalent DMSO-treated cultures as shown in Figure 4A. The mean fluorescence values were 15,766.67±721.88 msi (p<0.001), 13,566.67±578.31 msi (p<0.001), and 8166.66±176.38 msi (p<0.01) for 1,000, 500, and 300 µM chrysene, respectively, as compared to the respective DMSO-treated cultures. Cells treated with 100 µM chrysene did not show a significant increase in ROS/RNS level (3666.667±202.7 msi, p>0.05) as compared to DMSO-treated cultures (2066.667±120.18 msi). Values for untreated cells and DMSO-equivalent cultures of 1,000, 500, and 300 µM were 1860±124.9, 2366.67±233.3, 2166.667±176.4, and 2086.67+135.44, respectively. Pretreatment with 80 µM LA resulted in an 80.02% (14,466.67±785.99msi, p<0.001) decrease in ROS/RNS levels as compared to the 500-µM-chrysene-treated culture (2893.333±157.19 msi; Figure 4B).

### Mitochondrial membrane potential assay

**Figure 5:**
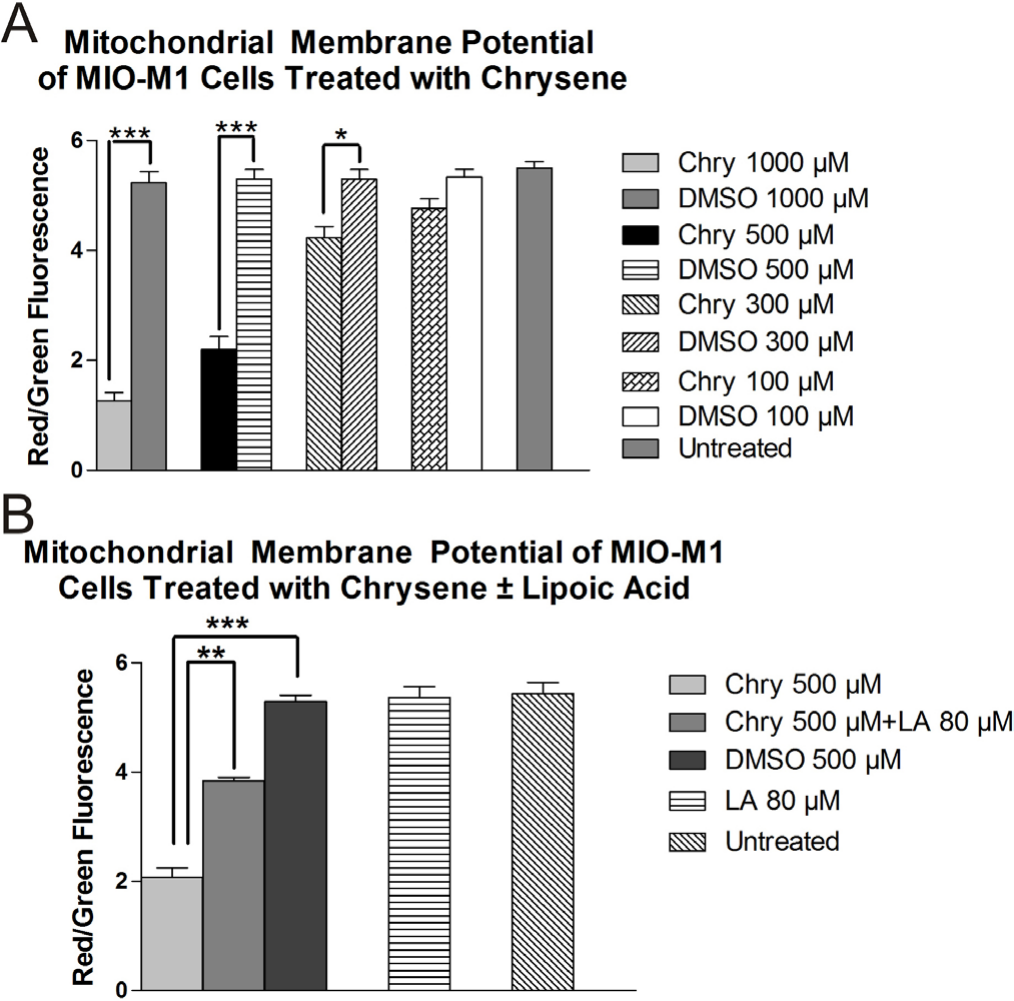
The effects of chrysene (Chry) on mitochondrial membrane potential (ΔΨm) in MIO-M1 cells. **A**: The MIO-M1 cells treated 24 h with 1,000 μM (***p<0.001), 500 μM (***p<0.001), and 300 μM (*p<0.05) chrysene showed lower ΔΨm compared to the cells treated with the dimethyl sulfoxide (DMSO)-equivalent. The 100-μM-chrysene-treated cells and DMSO-equivalent-treated cells showed similar levels of ΔΨm as the untreated cells. **B**: When the MIO-M1 cells were pretreated for 6 h with 80 μM lipoic acid (LA) and then 500 μM chrysene was added to the cultures for 24 h (Chyr 500 μM +LA 80 μM), the ΔΨm was restored partially compared to the 500-μM-chrysene-treated cells (**p<0.01). The 80 μM LA did not have any affect on the ΔΨm compared to untreated or DMSO-equivalent-treated cells. The assays for each concentration were run in triplicate repeats and the experiments repeated three times. Values are mean±standard error mean (SEM).

MIO-M1 cells treated for 24 h with 1,000, 500, and 300 µM chrysene showed significantly decreased ΔΨm as compared to their respective DMSO-treated cultures as shown in Figure 5A. The mean ΔΨm values were 1.27±0.15 (p<0.001), 2.2±0.23 (p<0.001), and 4.23±0.20 (p<0.05) for 1,000, 500, and 300 µM chrysene, respectively. Cells treated with 100 µM chrysene did not show a significant decrease in ΔΨm values (4.76±0.176, p>0.05) as compared to DMSO-treated cultures (5.33±0.145 msi). Values for untreated cells and DMSO-equivalent cultures of 1,000, 500, and 300 µM were 5.5±0.12, 5.23±0.2, 5.30±0.17, and 5.31±0.19, respectively. Pretreatment with 80 µM LA resulted in an 86% (3.84±0.05, p<0.001) increase in the ΔΨm value as compared to the 500-µM-chrysene-treated cultures (2.06±0.17; Figure 5B).

### Intracellular ATP levels

**Figure 6:**
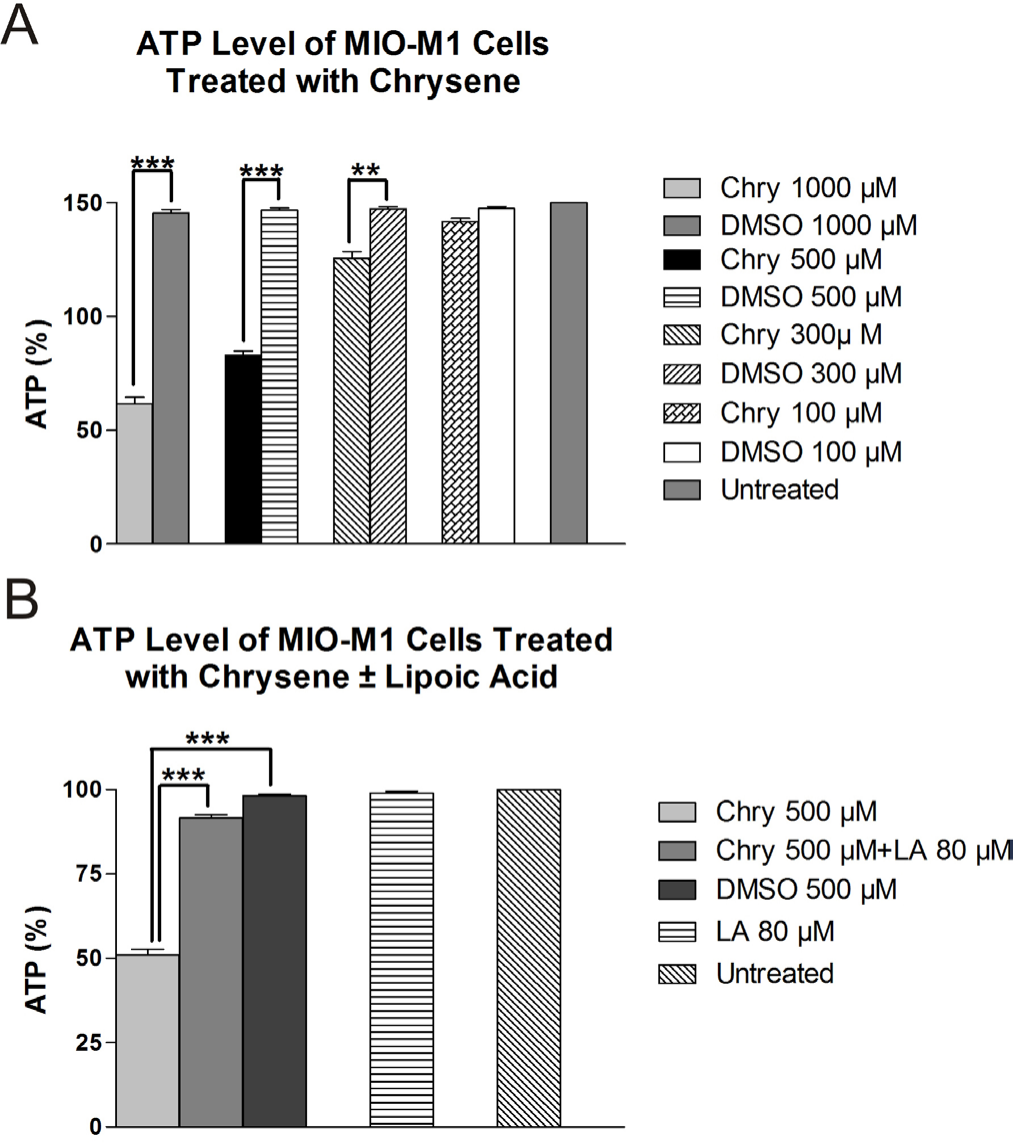
The effects of chrysene (Chry) on ATP production in MIO-M1 cells. **A**: The MIO-M1 cells exposed for 24 h to 1,000 μM, 500 μM, and 300 μM chrysene showed significantly lower ATP levels compared to dimethyl sulfoxide (DMSO)-equivalent-treated cells (***p<0.001, ***p<0.001, **p<0.01, respectively). The 100-μM-chrysene-treated cells had similar ATP levels to the DMSO-equivalent-treated and untreated cells. **B**: Pretreatment for 6 h with 80 μM lipoic acid (LA) significantly reversed the decline in ATP production caused by treatment with 500 μM chrysene (***p<0.001). The untreated cells, DMSO-equivalent-treated cells, and 80-μM-LA-treated cells had similar ATP production levels. The assays for each concentration were run in triplicate and the experiments repeated three times. Values are mean ± standard error mean (SEM).

Intracellular ATP levels in MIO-M1 cells decreased significantly after treatment for 24 h with 1,000, 500, and 300 µM chrysene as compared to their respective DMSO-treated cultures (Figure 6A). The mean percentage ATP levels were 41.0±2.08 (p<0.001), 55.3±1.2 (p<0.001), and 83.77±2.05 (p<0.01) for 1,000, 500, and 300 µM chrysene, respectively, as compared to the DMSO-treated cultures. Cells treated with 100 µM chrysene did not show a significant increase in ATP levels (94.66±0.88%, p>0.05) as compared to DMSO-treated cultures (98.33±0.6%). Values for untreated cells and DMSO-equivalent cultures of 1,000, 500, and 300 µM were 100±0, 97±1.15, 97.83±0.73, and 98.26±0.65, respectively. Pretreatment with 80 µM LA resulted in a 40.05% (91.5±1.15, p<0.001) increase in the ATP level as compared to the 500-µM-chrysene-treated cultures (51.0±1.73; Figure 6B).

## Discussion

In AMD, multiple cell types within the macula can be damaged. While the primary pathology involves the RPE cells, Bruch’s membrane, and the choriocapillaries [39], other cell types of the overlying neuroretina are also affected. In this study we wanted to focus on the retinal glial cells since they have an important supportive role for the health of RPE cells and the neuroretina. We used the human Müller cells as representative of the human retinal glial cells.

Human retina has three basic types of glial cells: Müller cells, astroglia, and microglia. Müller cells provide a supportive function to the neurons of the retina, including the photoreceptors, bipolar cells, and ganglion cells [40]. They also preserve the homeostasis of the retina by secreting growth factors and cytokines that maintain the outer blood–retinal barrier [41]. In AMD, these dysfunctional Müller cells are seen as accumulated lipid-bloated microglial cells in the subretinal spaces [42] and appear on fundoscopy to be similar to the drusen. With loss of the RPE cell barrier, choroidal neovascularization can occur. The Müller cells may be involved indirectly in the pathology of AMD since they are supportive of the outer blood–retinal barrier, which is damaged both in the early and late stages of AMD.

Recent studies have reported the progenitor properties of Müller cells. Müller cells in isolation display features similar to retinal progenitor cells as they can renew themselves and generate all of the neuronal cell types characteristic of retina [43,44]. Following retinal degeneration, progenitor cells derived from Müller cells differentiate into several different retinal cell lineages, which can support retinal regeneration in vivo [45,46]. However, because they are limited in numbers, these new retinal cells cannot completely replace damaged tissues [45]. From the above discussion, it becomes obvious that damage to the Müller cells can have a dramatic impact, both quantitative and qualitative, on the overall functioning of the macula.

Müller cells have additional functions after retinal degeneration [47,48], including re-entering the cell cycle and producing neuroprotective growth factors. Once these cells are activated, they become involved in the formation of glial scars, which occurs in late phases of retinal degeneration [47]. The proliferation of Müller cells depends on the activation of the Wnt/β-catenin (Wnt) pathway and the sonic hedgehog (Shh) pathway [49]. The scar tissues seen clinically in some of the end stages of wet AMD are a direct result of this Müller cell activity.

We first investigated the effect of low concentrations of chrysene (20, 30, 40, and 80 µM) on the CV of MIO-M1 cells after 24 h of exposure. Chrysene had no apparent effect on CV at these concentrations. However, there was a significant loss of CV after 7 days (chronic) treatment with 30 µM chrysene. This damaging effect on cells demonstrates that chrysene is toxic to MIO-M1 cells and could be a contributing factor to MIO-M1 cell degeneration due to cigarette smoking. MIO-M1 cells after 24 h of treatment with 1,000, 500, and 300 µM chrysene showed a significant concentration-dependent loss of CV as compared to their respective control. This kind of toxicity has also been shown in other cell types treated with other components found in cigarette smoke. Patil et al. have shown a significant decrease in CV in HMVEC and rat neurosensory retinal (R28) cells treated with 10^−2^ M nicotine [26]. The HMVEC cultures underwent nonapoptotic loss of cell viability and the R28 cells had decreased viability through an oxidative, noncaspase-dependent, apoptotic pathway [26]. In another study, the RPE cells were damaged through oxidative and mitochondrial dysfunction pathways after exposure to acrolein [27]. Another toxin, B(e)P, caused a dose-dependent decrease in the CV of ARPE-19 cells [24]. Both chrysene and B(e)P are PAHs, but B(e)P has five fused benzene rings, while chrysene has four fused benzene rings. Therefore, it is not surprising that both compounds would have damaging effects on cultured cells.

In the present work we elaborated on the mechanism of toxicity on MIO-M1 cells after treatment with 1,000, 500, and 300 µM chrysene. MIO-M1 cells treated with these concentrations of chrysene for 24 h had significantly increased caspase-3/7 activity. DNA fragmentation analysis with 500 µM chrysene also showed DNA banding that laddered in approximately 200-bp increments. Both assays are consistent with apoptosis as the mechanism of cell death. Necrosis also played a role in cell death but only at the highest concentration of chrysene (1,000 µM). Therefore, in this system both apoptosis and necrosis might be the major mechanisms of cell death. In other assays, MIO-M1 cells treated for 24 h with 1,000, 500, and 300 µM chrysene showed significantly increased ROS/RNS and decreased ΔΨm and ATP levels, suggesting involvement of oxidative stress and mitochondrial dysfunction in chrysene-induced toxicity. Mitochondria are the main generation site of oxidants and are also a target of oxidants [50]. The constant generation of superoxide and hydroxyl radicals by mitochondria causes continuous oxidative stress. Free radicals produced during oxidative metabolism [51] can damage mitochondrial DNA (mtDNA) [52] and may increase exponentially with age. As mtDNA damage accumulates, the electron transport chain is less efficient, leading to greater free radical (superoxide) production [53]. The vicious cycle of oxidation, depletion of cellular antioxidants, such as glutathione [54], and exacerbation of mitochondrial damage may be responsible, in part, for cellular decay. With our data we speculate that damage to the mitochondria could be the initiating event in the cascade leading to eventual apoptotic or necrotic death.

Several studies have reported on caspase activation, necrosis, oxidative stress, and mitochondrial dysfunction as a cell-death mechanism. Human RPE cells after treatment with B(e)P underwent apoptosis as shown by elevated activities of caspase-3/7, caspase-8, caspase-9, and caspase-12 [24]. Müller cells following treatment with indocyanine green dye showed that cell death and morphological changes were concentration and time dependent [55]. Human monocytic leukemia U937 cells after treatment with 2-tert-butyl-4-hydroquinone (a phenolic antioxidant used as a food additive) and 2-tert-butyl-1,4-benzoquinone (its metabolite) showed apoptotic and necrotic effects as demonstrated by elevated caspase activities, DNA fragmentation, decreased ATP, and elevated LDH levels [56]. Renal epithelial cells after exposure to iodoacetamide (a prototypical alkyating agent) showed apoptosis and necrosis [57]. Human endothelial cells and monocytes exposed to tobacco smoke and benzo[a]pyrene smoke rapidly induced complex oxidant-mediated stress responses, loss of mitochondrial membrane potential, and apoptosis or necrosis at higher concentrations that caused cell death [58]. In another report, Müller cells treated with methanol showed toxicity by ATP depletion [59]. These studies indicate that the mode of cell death differs depending on the toxin used.

Besides exploring the toxic effects of chrysene on MIO-M1 cells, we have also studied the reversal of chrysene-induced toxicity using LA. In some published reports from our laboratory, pretreatment of retinal cells with different inhibitors before exposure to toxins have shown protective effect on cells. For example, pretreatment of ARPE-19 cells with memantine, resveratrol, and genistein significantly inhibited loss of CV after exposure to B(e)P. However, ARPE-19 cells pretreated with epicatechin did not significantly reverse CV [25]. In other studies using R28 cell cultures, increased caspase-3 activity caused by 7-ketocholesterol (toxic element from oxidized low-density lipoproteins) was significantly reduced after pretreatment with epicatechin [28]. The diversity and disparity in the protection of retinal cells may be due to the nature of the toxin and type of cell lines.

MIO-M1 cultures pretreated with 80 µM LA showed reversal of caspase-3/7, ROS/RNS, ΔΨm, and ATP effects induced by chrysene. This finding strongly suggests that LA offers protection to cells against caspase-dependent oxidative stress and mitochondrial dysfunction. LA is a natural metabolic antioxidant [60] that is easily absorbed, crosses the blood–brain barrier, and reaches peak levels in the central nervous system, retina, and peripheral nerves within 0.5 h of oral administration [61]. Inside the mitochondria of the cells, mitochondrial α-keto acid dehydrogenase complexes [61] reduce LA into another more potent antioxidant—dihydrolipoate. Both LA and especially dihydrolipoate regenerate other antioxidants, such as vitamin C and vitamin E, through redox cycling and raise intracellular glutathione levels [61]. LA administration increases intracellular glutathione levels by 30–70% in cell culture and in vivo studies [62,63]. LA targets mitochondria, protects mitochondria from oxidative damage, and improves mitochondrial function. The mechanism of protection includes preventing the generation of oxidants, scavenging free radicals and iron chelating [64], inhibiting oxidant reactivity, elevating cofactors of defective mitochondria to enhance antioxidant defense system [65], and protecting enzymes from further oxidation. LA repairs oxidative damage to lipids and proteins/enzymes through activation of the phase 2 enzyme system and increases mitochondrial biogenesis and improves its function [51].

LA is available as a food supplement in grocery stores in Europe and the USA. LA is clinically safe and is considered to have a beneficial effect in several disorders, such as cerebral ischemia–reperfusion, excitotoxic amino acid brain injury, mitochondrial dysfunction, diabetes and diabetic neuropathy, inborn errors of metabolism, and other causes of acute or chronic damage to brain or neural tissue [61]. Several studies using cell culture and disease models in animals have shown favorable effects of LA. LA added to cultural rat hippocampal neurons reversed cell damage induced by glutamate [66]. Pretreatment of human fetal retinal pigment epithelial cells with LA blocked the ROS production and apoptosis and increased the mitochondrial membrane potential due to a chemical oxidant, *tert*-butylhydroperoxide [67]. Treatment with LA, creatine, nicotinamide, and epigallocatechin gallate protected ganglion cell death due to apoptosis, oxidative stress, and mitochondrial dysfunction in rats [68]. Treatment with thioctic acid and dihydrolipoic acid demonstrated neuroprotection against N-Methyl-D-aspartic acid (NMDA) and malonic acid lesions of striatum in rats [69]. LA showed caspase-dependent and -independent inhibition of cell death against pilocarpine-induced seizures in rats [70]. LA demonstrated anti-apoptotic and neuroprotective effects on spinal cord ischemia–reperfusion in rabbits [71]. LA showed protective effects in D-galactose-induced memory loss, neurodegeneration, and oxidative damage in mice [72].

Administration of LA with other agents has shown synergistic effects in animals as well as in patients with Alzheimer disease (AD). Co-administration of LA and vitamin C inhibited oxidative stress in rats exposed to chronic arsenic toxicity [73]. LA given with vitamin E has shown additive effects against lipid peroxidation in several pathological models for neurologic functions, glial reactivity, and neuronal remodeling in rats [74]. Administration of LA and N-acetyl cysteine has shown decreased mitochondrial-related oxidative stress in fibroblasts from AD patients [75]. It has been suggested that combined therapy of LA with antioxidants could greatly diminish the progression of AD disease [76,77]. Reports from 43 patients that were observed for 48 months provided supportive findings that LA treatment could provide neuroprotection for AD patients [78].

LA has been shown to be beneficial in some eye disorders and diabetes models. Long-term administration of LA inhibited diabetic retinopathy by reversing mitochondrial dysfunction and retinal capillary cell death in rat [79,80]. The combination of LA with lutein, zeaxanthin, and l-glutathione protected photoreceptor degeneration in animal models for retinitis pigmentosa [81]. LA, acetyl-L-carnitine, nicotinamide, and biotin have been found to improve immune dysfunction in type 2 diabetic rats [82]. The numerous reports detailed above are consistent with our findings that LA can be protective against chrysene-induced toxicity in MIO-M1 cells.

In summary, based upon our data we suggest that the mechanism of cell death of MIO-M1 cells after chrysene treatment involves both apoptosis and necrosis. Lipoic acid, a natural antioxidant, could reverse chrysene-induced apoptosis, oxidative damage, and improve mitochondrial function in Müller cells. Therefore, administrating LA and its derivatives to aged people may be an effective strategy for improving or delaying neurodegenerative disorders, such as AMD.
